# Sirtuins and Immuno-Metabolism of Sepsis

**DOI:** 10.3390/ijms19092738

**Published:** 2018-09-13

**Authors:** Xianfeng Wang, Nancy L. Buechler, Alan G. Woodruff, David L. Long, Manal Zabalawi, Barbara K. Yoza, Charles E. McCall, Vidula Vachharajani

**Affiliations:** 1 Departments of Anesthesiology, Wake Forest School of Medicine, Winston-Salem, NC 27157, USA; xwang@wakehealth.edu (X.W.); nbuechle@wakehealth.edu (N.L.B.); agwoodru@wakehealth.edu (A.G.W.); 2 Departments of Internal Medicine, Wake Forest School of Medicine, Winston-Salem, NC 27157, USA; dllong@wakehealth.edu (D.L.L.); mzabalaw@wakehealth.edu (M.Z.); byoza@wakehealth.edu (B.K.Y.); 3 Departments of Surgery, Wake Forest School of Medicine, Winston-Salem, NC 27157, USA

**Keywords:** sepsis, septic shock, hyper-inflammation, immunosuppression

## Abstract

Sepsis and septic shock are the leading causes of death in non-coronary intensive care units worldwide. During sepsis-associated immune dysfunction, the early/hyper-inflammatory phase transitions to a late/hypo-inflammatory phase as sepsis progresses. The majority of sepsis-related deaths occur during the hypo-inflammatory phase. There are no phase-specific therapies currently available for clinical use in sepsis. Metabolic rewiring directs the transition from hyper-inflammatory to hypo-inflammatory immune responses to protect homeostasis during sepsis inflammation, but the mechanisms underlying this immuno-metabolic network are unclear. Here, we review the roles of NAD+ sensing Sirtuin (SIRT) family members in controlling immunometabolic rewiring during the acute systemic inflammatory response associated with sepsis. We discuss individual contributions among family members SIRT 1, 2, 3, 4 and 6 in regulating the metabolic switch between carbohydrate-fueled hyper-inflammation to lipid-fueled hypo-inflammation. We further highlight the role of SIRT1 and SIRT2 as potential “druggable” targets for promoting immunometabolic homeostasis and increasing sepsis survival.

## 1. Introduction

Sepsis and septic shock kill over 200,000 people each year and are the most expensive conditions in the U.S. with an annual cost of over $20 billion [1]. Globally, 20–30 million patients are diagnosed and over eight million lives lost each year with sepsis and septic shock [2]. The early hyper-inflammatory phase transitions within hours to the late/hypo-inflammatory and profoundly immunosuppressive phase [3]. While nearly one third die from early sepsis, the majority of sepsis-related deaths occur during late sepsis [4]. The late/hypo-inflammatory phase is characterized by multiple organ failure and endotoxin tolerance [3,5]. Over 30 different clinical trials, almost all targeting the early/hyper-inflammatory phase of sepsis, failed to improve mortality, highlighting the knowledge and treatment gaps [6]. Evidence suggests that these phenotypic changes in immune cells accompany the change in substrate utilization for energy [7]. A block in glucose uptake and glycolysis with repressed mitochondrial respiration contributes to hypo-inflammation [8,9].

Sirtuins (SIRTs) are the mammalian homologues of silencer regulator of information (Sir2p) genes first described in yeast [10]. There are seven sirtuins (SIRT1-7) dispersed amongst different cell compartments. A distinct feature of sirtuin family members is their NAD+-dependent deacetylase and ADP ribosylation enzymatic properties. SIRTs are the key regulators of inflammatory stress response in immune and non-immune cells [11,12]. Mounting evidence strongly implicates the role of sirtuins in epigenetically directing the course of sepsis by coordinated metabolic, immune and bioenergetics reprogramming (immuno-metabolism) [7,8,12,13].

Although Sir2 was discovered as a metabolic and NAD+ redox sensor and epigenetic regulator, the mammalian SIRTs broadly reprogram metabolism, immunity and mitochondrial bioenergetics [7,9,14] and guard cell-homeostasis [15]. This review focuses on the role of different sirtuins in sepsis and their potential for small molecule targeted treatments that modify homeostasis control by this family.

## 2. Immunologic Dysfunction in Sepsis

**Figure 1:**
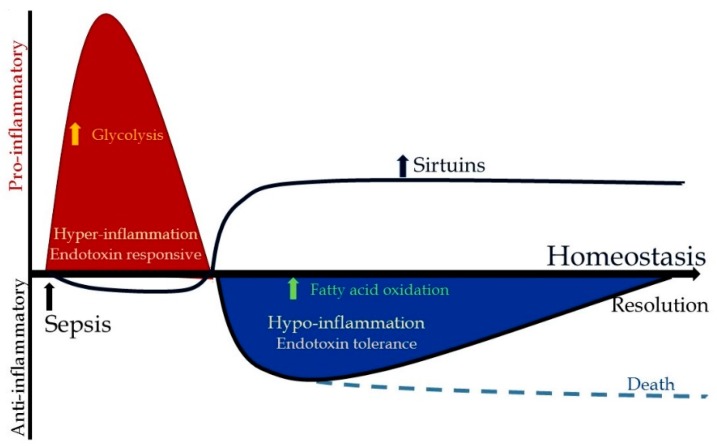
Immune response to sepsis: sepsis inflammation in immune cells transitions from the hyper-inflammatory to the hypo-inflammatory phase. There is increased glycolysis in the endotoxin-responsive hyper-inflammatory phase, while the endotoxin-tolerant hypo-inflammatory phase is associated with increased fatty acid oxidation in immune cells.

As the early hyper/inflammatory phase of sepsis transitions to a late/hypo-inflammatory phase, there is a profound departure from the homeostasis depicted in Figure 1; a model conceptually similar to the reported literature [16]. As the name suggests, the early hyper-inflammatory phase is associated with pro-inflammatory response, intended to kill invading pathogens. The late/hypo-inflammatory phase, in contrast, intended to be cytoprotective towards tissue and organs, is associated with immunosuppression and inability to clear infections [17,18]. Clearly, the therapeutic options are vastly different during early vs. late phases of sepsis [4]. One can speculate that phase-dependent therapeutic strategies during two phases, namely the anti-inflammatory therapy during early/hyper-inflammation and the pro-inflammatory strategy during hypo-inflammation in order to restore/return to homeostasis would be beneficial. However, recognition of the exact phase of sepsis and deployment of phase-specific therapies aimed at restoration of homeostasis are of utmost importance.

## 3. Metabolic and Bioenergetic Changes during Sepsis

A better understanding of the mechanisms responsible for transition from the hyper- to the hypo-inflammatory phase will likely lead to a newer phase of specific therapeutic targets and improve sepsis survival. Cell, animal and human proof-of-principle models suggest temporally controlled metabolic shifts in immune cells during this transition [18,19,20]. To understand these metabolic shifts, an insight into the changes in the microenvironment during sepsis inflammation is important.

### 3.1. Microenvironment Contributions

In contrast with the normal tissue microenvironment replete with oxygen/nutrients, the inflammatory milieu creates a competition for these. The invading microorganisms including bacteria [21,22] and viruses [23,24] utilize glucose for fuel and glycolysis-related enzymes for their replication. As outlined below, the effector immune cells also need glucose, for phagocytosis and bacterial killing. This means the effector immune cells must compete with pathogens for glucose for their effective immune function [21,22].

### 3.2. Metabolic Changes during Hyper-Inflammation

During the early/hyper-inflammatory phase of sepsis, the immune and non-immune cells such as dendritic cells, macrophages, natural killer cells, neutrophils and epithelial cells [19,25,26,27,28], the early responders to infecting pathogens, have three specific requirements: (1) energy (2) “pathogenic killing capacity” and (3) ability to rapidly regenerate to support pathogenic killing in the face of increased apoptosis of immune cells. Immune cells, similar to proliferating cancer cells, fulfil these three requirements by undergoing aerobic glycolysis also described as the “Warburg effect” [29]. Otto Warburg observed that the proliferating cancer cells need energy (ATP) and nucleotide synthesis for rapid regeneration. To fulfil these, the cancer cells preferentially undergo glycolysis under aerobic conditions. Evidence suggests that similarly, the effector immune cells also undergo aerobic glycolysis as described below [29,30].

(1) Energy requirements: It has been known since the 1960s that the effector immune cells need energy in the form of ATP for phagocytosis [31,32,33,34]. Furthermore, it was also noted that while disruption of glycolysis affects the ability of immune cells to perform phagocytosis, inhibition of the cytochrome system and disruption of the TCA cycle does not, indicating that ATP generation during phagocytosis is dependent on glycolysis [32,35]. To achieve this, glucose enters immune cells via upregulation of GLUT1 [36], and GLUT1 expression increases during hyper-inflammation [7,36,37,38]. Once inside the cell, the glucose is converted to pyruvate via a series of enzymatic processes with hexokinase, phosphofructokinases and glyceraldehyde-phosphate dehydrogenase as rate-regulating enzymes [20]. In mammalian cells, pyruvate, the pre-mitochondrial product of glycolysis, metabolizes to lactate or enters mitochondria after decarboxylation by the pyruvate dehydrogenase complex (PDC) to form acetyl coenzyme A (acetyl-CoA). Acetyl-CoA feeds into the citric acid cycle and electron transport chain in the mitochondria to generate ATP. Extra-mitochondrial glycolysis nets two ATP, and intra-mitochondrial oxidative phosphorylation from glucose generates 36 molecules of ATP per molecule of glucose.

While efficient in ATP-generation, oxidative phosphorylation is slower than glycolysis. Glycolysis, on the other hand, although inefficient for ATP generation per molecule of glucose, can be ramped up rapidly. Thus, glycolysis is able to meet the anabolic “energy requirement” of innate immune cells by simply utilizing more molecules of glucose to undergo aerobic glycolysis [8,20,39,40]. Moreover, the immune cells reciprocally reduce oxidative phosphorylation during the early hyper-inflammatory response of sepsis [29,30,40,41]. Increased aerobic glycolysis, decreased oxidative phosphorylation and increased NADPH oxidase that generates reactive oxygen species in response to stress in immune cells was reported as early as 1984 [41].

**Figure 2:**
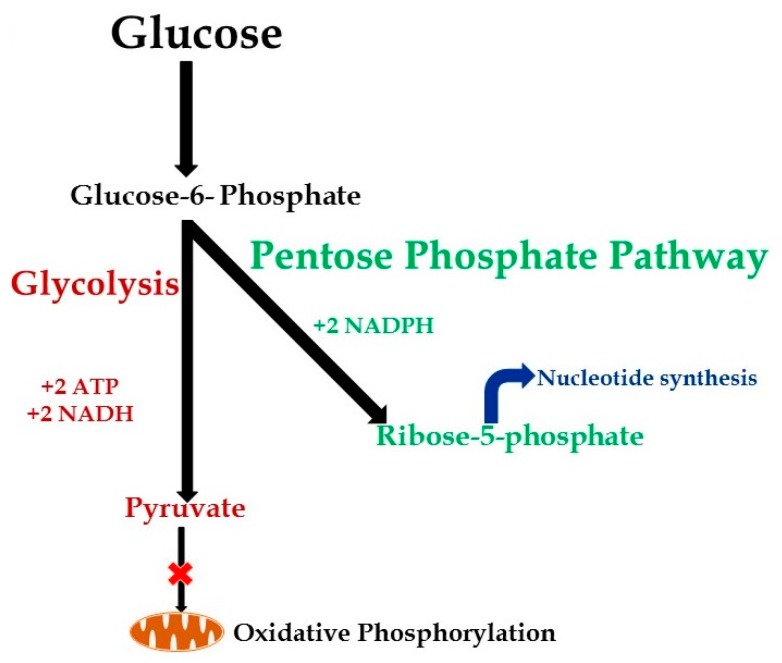
Aerobic glycolysis in activated immune cells: Activated immune cells undergo aerobic glycolysis; there is inhibition of oxidative phosphorylation in these cells. In addition, there is a selective increase in the pentose phosphate pathway in these cells resulting in ribose-5-phosphate to facilitate nucleotide synthesis to support cell proliferation.

(2) Pathogen-killing capacity: Immune cells resist infection by killing or containing microorganisms. Reactive oxygen species support the “killing capacity” of immune cells, particularly phagocytes [42,43,44]. Activated innate and adaptive immune cells increase glycolysis and divert glucose-6-phosphate into the pentose phosphate pathway. The pentose phosphate pathway fuels NADPH to activate NADPH oxidase and generate the ROS-dependent “killing capacity” needed by the immune cells [20,41,45]. Moreover, evidence also suggests that glucose-6-phosphate dehydrogenase (G-6PD) is essential for neutrophil extracellular trap formation to further assist with phagocytosis and killing of pathogens [46]. Deficiencies in G-6PD increase susceptibility to and mortality by sepsis possibly due to decreased phagocytosis via impairment of the pentose phosphate pathway and NADPH activity as a result [47,48,49,50].

(3) Rapid immune cell regeneration: Immune cells may die by apoptosis during early sepsis; profound lymphopenia is observed in sepsis and other acute inflammatory injuries [51,52]. Evidence suggests that T lymphocytes from septic shock patients exhibit decreased glycolysis, as well as glucose uptake along with lymphopenia via the mTOR-HIF-1α pathway; IL-7 treatment restores immuno-metabolic defects and the ability for T cell expansion [36]. Naive T cells depend on OXPHOS for their metabolism. Evidence suggests that CD4+ cells depend on GLUT1 receptors, while CD8+ cells depend on upregulation of other members of GLUT family, GLUT3 and GLUT6, expression on their surface for glucose uptake; however, the importance of GLUT3 and GLUT6 in regulating CD8+ cell function is unclear [53]. The immune-repressor T-regulatory (T-reg) cells seem to not depend upon glycolysis, but rather fatty acid oxidation for their energy requirement and expansion [53]. Sepsis-related apoptosis is induced during increased phagocytosis and ROS production [54]. While it is unclear whether neutrophil apoptosis is protective or harmful to the host [55,56], early immune cell activation and increased cell death require a marked increase in cell biomass and in lymphocyte replication to rapidly replete those cells. Glucose-6-phosphate generated during aerobic glycolysis feeds into the pentose phosphate pathway (Figure 2) to generate ribose phosphate, a building block necessary for nucleic acid synthesis in a manner similar to proliferating cancer cells [20].

Thus, glycolysis fulfils all three “needs” of the activated immune cell during the hyper-inflammatory phase: the energy requirement (ATP), pathogenic killing capacity (NADPH) and nucleotide synthesis for cell proliferation (Figure 2). Glycolysis also fuels fatty acid synthesis and protein synthesis.

### 3.3. Metabolic Changes during Hypo-Inflammation

The early hyper-inflammatory phase of sepsis, while supporting pathogenic clearance, is cytotoxic for the host immune cells and tissue/organs. Energy depletion for biosynthetic processes, cell injury and mass cell death processes cause immune cells to abandon resisting infection and environmental threats to enter a “cell hibernation” mode accompanied by the hypo-inflammatory phase during late sepsis [57]. This transition from hyper- to hypo-inflammation is seen in macrophages and T cells [58,59]. The pro-inflammatory macrophages transition to the endotoxin-tolerant hypo-inflammatory phenotype during this phase. Interestingly, while the cytokine profile of these macrophages changes from the hyper- to hypo-inflammatory phenotype, these cells are fundamentally different from the traditional “M2” type macrophages. These hypo-inflammatory macrophages fail to recruit chemokines including chemokine (C-C motif) ligand 17 (CCL17), CCL22, and CCL24; chemokines important for T cell recruitment. This adds to the inability of the host to stimulate adaptive immune response, adding further to the sepsis-induced immunosuppression [60]. These macrophages exhibit endotoxin tolerance, a well-accepted indicator and a marker of hypo-inflammation/immunosuppression [19,61,62]. At the cellular level, this process is not all or none, but the ratio or net polarity of pro vs. anti-inflammatory determines the clinical phenotype during sepsis. This “survival of the fittest” state that resembles both starvation and suspended animation is accompanied by a shift in the substrate utilization of immune cells from glucose to fatty acid oxidation.

Fatty acid oxidation: Substrate selection for energy fuel and ATP generation is critically important for understanding the immuno-metabolism and its contribution to sepsis phases and outcomes. Lipolysis of imported lipids or stored triglycerides provide fuel for endotoxin-tolerant hypo-inflammatory immune cells unless they are severely starved as in sepsis; the sepsis starvation state mutes glucose and fatty acid mitochondrial fueling [8].

Free fatty acids enter the macrophages/monocytes with the help of fatty acid transporters such as CD36. After cellular entry, the long-chain fatty acids must be fused with carnitine, by CPT-I, to form acyl-carnitine molecules that can enter the mitochondrion. Inside the mitochondrion, the acyl-CoA is freed from the carnitine molecule, and β-oxidation is initiated. Acetate molecules from the fatty acid chain combined with CoA form acetyl-CoA, which then enters the TCA cycle, fusing with oxaloacetate to form citrate [20].

Increased levels of CD36 and CPT-I during hypo-inflammation in cell models of sepsis and leukocytes from septic mice support the notion that there is increased fatty acid uptake in cytosol and transfer into mitochondria [7,63,64]. Evidence suggests that STAT6 and PGC-1 prime macrophages for alternative activation to mute pro-inflammatory phenotype, thus linking mitochondrial oxidative metabolism with the anti-inflammatory phenotype in immune cells [64]. SIRT1 lies proximal to PGC-1 in this endotoxin-tolerant hypo-inflammation supporting pathway [7]. PGC-1 knockdown decreases fatty acid oxidation while increasing glycolysis and the pro-inflammatory effector properties of macrophages [64].

A seminal non-biased metabolomics study in over 1000 patients with sepsis [65] and in non-human primates [66] suggests a strong relationship between the fatty acid oxidation pathway and sepsis survival. These studies show that dysregulated fatty acid oxidation with increased long and short chain acyl carnitine fatty acids within 48 h of sepsis onset are predictors of sepsis survival. Specifically, six carnitine metabolites increased in survivors, while 16 carnitine esters and four fatty acids were elevated in non-survivors of sepsis. Interestingly, fatty acid CPT1 transporters decreased in non-survivors, further supporting this relationship [65]. Increased CD36 expression levels were observed during apoptosis-induced immunosuppression in peripheral blood mononuclear cells [67]. Together, these data support abnormal management of glucose and lipid nutrient substrates during life-threatening sepsis with profound immune suppression and organ failure.

Mitochondrial dysfunction: Investigators are intrigued by the lack of structural damage to tissue/organs in patients with sepsis and septic shock despite functional dysregulation. The absence of severe organ cell damage during sepsis except mitochondrial structural changes (e.g., liver) suggests a crucial role for metabolic dysregulation that impairs cellular bioenergetics. Like starvation and hibernation, oxygen consumption and nutrient anabolism decrease during sepsis [68]. Thus, increased oxygen consumption rates for anabolism during the hyper-inflammatory phase switch to decreased oxygen consumption during the hypo-inflammatory phase in animal, human and cell models [7,69]. If the cellular metabolism feeding biomass expansion and cellular replication continue without enough ATP (an imbalance in energy demand and supply), this apparent ATP deficit can activate apoptotic pathways. To avoid this, the hibernating cells during the hypo-inflammatory phase of sepsis compensate by switching off the metabolic processes that are not directly involved in cell survival [70]. The rebound increase in oxygen consumption occurs in resolving sepsis and ischemia reperfusion injury [69,70].

It is critical to understand how the immuno-metabolism transitions between these immunometabolic and bioenergy phenotypes during life threatening sepsis. As introduced in this review, the SIRT family members are critical for this shift in immuno-metabolic phenotype.

## 4. Sirtuins and Sepsis Immuno-Metabolism

**Figure 3:**
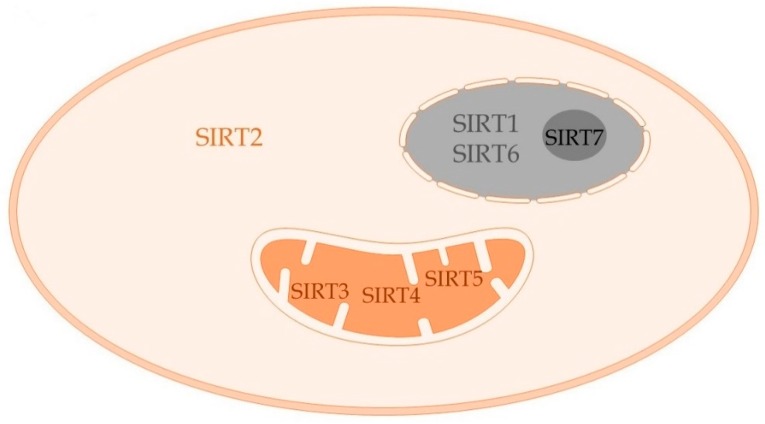
Sirtuin expression in immune cells: The seven sirtuins SIRT1–7, the NAD+ sensors, are dispersed throughout subcellular compartments of cells. SIRT1 and SIRT6 are nuclear, SIRT7 is nucleolar, and SIRT3, SIRT4 and SIRT5 are localized in mitochondria, while SIRT2 is predominantly cytosolic. Each of these sirtuins have their own unique targets that define their biological activity as described in the text.

Sirtuins are a highly conserved family of proteins first described in yeast and extensively studied in aging literature. Sirtuins are known for anti-inflammatory and anti-oxidant properties. The seven sirtuins (SIRT1–7) belong to the class III histone deacetylase family of enzymes [71,72,73]. These seven sirtuins, considered the “energy sensors” of cell, disperse throughout the cell compartments [74,75]. With a predominant function of deacetylation, each of the sirtuins has its own deacetylation targets that determine their unique biological function. As shown in Figure 3, SIRTs 1, 6 and 7 are nuclear; SIRTs 3, 4 and 5 mitochondrial; and SIRT2 is primarily cytosolic [76]. Sirtuins are NAD+-dependent deacetylases; the expression of each of these sirtuins seems to be controlled by the NAD+ concentration within the compartment to which they belong [77].

Increased glycolysis during the hyper-inflammatory phase in addition to decreased oxidative phosphorylation lead to accumulation of NAD+. The two components of NAD+ accumulation that modulate “energy sensing” in cells are NAD+ biosynthesis keeping pace with the metabolic demands of cells and NAD+-dependent sirtuin levels that execute several metabolic functions of cells such as gluconeogenesis and fatty acid oxidation [78].

Sirtuins, due to their unique biological functions, deacetylate various histone and non-histone proteins and lead to a sustained anti-inflammatory phenotype, as described in the following sections. Specifically, SIRT 1, 2, 3, 4 and 6 are implicated in sustained hypo-inflammatory immune response in sepsis; SIRT 5 and 7 have a potential role that has been largely unexplored. Table 1 summarizes the available literature regarding the role of each of these sirtuins in specific immune cells and their mechanisms of action during sepsis inflammation. The detailed role of each of these sirtuins is described in the following sections.

### 4.1. SIRT1

SIRT1 is the most extensively studied sirtuin, especially its role in aging. Nearly two decades ago, Sir2p was shown to prolong longevity in yeast [83]. The interest in sirtuins grew even further when Sir2 mammalian orthologues emerged as NAD+-dependent deacetylases and/or ADP-ribosyltransferases [84,85]. In addition to its role in controlling lifespan (aging), stem cell development, cell differentiation and autophagy, SIRT1 emerged as a key regulator of inflammation when it was discovered that it deacetylates and deactivates NFĸB p65 and consequently the anti-inflammatory property during hypo-inflammation [86]. This property requires increased nuclear NAD+ production and accumulation as hyper-inflammation begins to peak. The role of NAD+ and SIRT1 deacetylation activation as an inflammation rheostat emerged when multiple studies reported that obesity and other chronic pro-inflammatory states are associated with reduced NAD+, NAMPT and SIRT1 levels in monocytes and other immune cells. In contrast, low calorie intake and exercise increase NAD+, NAMPT and SIRT1 mRNA and protein levels and repress NFĸB p65 activation [85,86]. The importance of the NAD+ world has emerged [78,87].

Increased expression/activity of SIRT1 reprograms the balance of glucose and fatty acid metabolism to support the immunometabolic homeostasis of cells. This axis of metabolism and its link to inflammation and immunity controls anabolic nutrient selection for increased glucose flux and glycolysis, which parallels target of rapamycin Complex 2 (TORC2) activation to increase protein synthesis and the pentose phosphate pathway to build nucleic acids [8]. Levels of SIRT1 are low during this anabolic state. As inflammation progresses, increases in NAD+, NAMPT and SIRT1 expression and activation counter anabolism by supporting a switch that selects fatty acids over glucose as a dominant substrate for energy along with glutamine [7]. SIRT1 supports the anti-inflammatory lipolytic rather than glycolytic substrate for immune cells by regulating peroxisome proliferator-activated receptor-gamma coactivator-α (PGC-1α) [88]. Thus, anti-inflammatory homeostasis conditions show increased NAD+ levels and repressed glucose uptake with increased lipolysis and fatty acid oxidation to enter a catabolic state of cell survival. Cell survival is further supported by SIRT1’s effect on decreasing apoptosis and supporting autophagy to derive energy for cell survival [85].

SIRT1 regulates immunometabolic polarity during the hyper-inflammatory and hypo-inflammatory phases of sepsis. Using THP-1 pre-monocytic cells with LPS stimulation and peripheral blood mononuclear cells from sepsis patients, we showed that during the hypo-inflammatory phase of sepsis, increased fatty acid oxidation with decreased glycolysis occurs with increased SIRT1 levels. Interestingly, SIRT1 protein levels decreased during the early/hyper-inflammation of sepsis [14]. Mechanistically, we have shown that the anti-inflammatory properties of SIRT1 are via deacetylation and deactivation of NFĸB p65 with decreased expression of NFĸB p65-dependent pro-inflammatory cytokine/chemokine and adhesion molecule expression in immune and endothelial cells [79]. When this anti-inflammatory phenotype is prolonged without timely return to homeostasis, immunosuppression of hypo-inflammation with the inability to clear pathogens ensues [19]. We showed that SIRT1 inhibition during hypo-inflammation increased the pro-inflammatory phenotype and bacterial clearance, i.e., reversed hypo-inflammation, as described in the section on therapeutic targeting of the sirtuin pathway below [19]. We have also shown that SIRT1 directly or indirectly influences the expression of SIRT 3 and 6 in sepsis [7,9], as reviewed in the respective sections.

### 4.2. SIRT2

SIRT2, far less studied than SIRT1, is emerging as distinctly important in obesity and inflammation, including obesity associated with sepsis. Like other family members, SIRT2 is ubiquitously expressed, especially in highly metabolically-active tissue such as brain, liver, adipose tissue, kidneys, pancreas and muscle [89,90,91]. SIRT2 is the most predominantly expressed sirtuin in the adipocytes and adipose tissue [90,92]. SIRT2 is a nutritional sensor in the adipose and immune cells, and like SIRT1, caloric restriction increases and hyper-nutrition and high fat diets decrease SIRT2 expression in white adipose tissue of mice [90,93]. Originally described as a tubulin deacetylase, similar to other SIRT family members, SIRT2 deacetylates histone and non-histone proteins [89,94].

While described as a predominantly cytosolic protein, SIRT2 translocates to the nucleus under cellular stress [94,95]. SIRT2 has a critical role in cell cycle regulation. SIRT2 shuttles to the nucleus during mitotic stress, where, due to its preference to histone H4 lysine 16 (H4K16), it regulates chromosomal condensation gene muting during mitosis [94]. Although important in regulating the cell cycle, the role of SIRT2 in tumorigenesis is unclear due to contradictory reports of SIRT2 knock out mice presenting with spontaneous tumors and SIRT2 inhibition prolonging tumor progression [96,97]. SIRT2 protein is highly expressed in brain and spinal cord; its role in inflammation and redox-associated neurodegenerative diseases such as Huntington’s disease and Parkinson’s disease is reported. SIRT2 inhibition is neuroprotective in these two conditions [98,99]. However, SIRT2 deficiency did not alter the progression of the disease, raising questions regarding the neuroprotective role of SIRT2 inhibition in Huntington’s disease [100].

SIRT2 senses and regulates metabolism, including use of glucose and fatty acids as substrates for oxidative bioenergetics. Pyruvate kinase catalyzes the final step of glycolysis, dephosphorylation of phosphoenolpyruvate to generate pyruvate. Its isotype, PKM2, is expressed abundantly in proliferating cells including tumors [101]. SIRT2 deacetylates PKM2 to enhance its tetramerization [101]. Evidence suggests that PKM2 in its tetrameric form has high affinity to phosphoenolpyruvate converting, generating pyruvate; the dimeric form does not [102]. Consequently, the tetrameric form of PKM2 supports the TCA cycle, while dimeric/monomeric forms support the Warburg effect [103,104]. We have recently shown that direct oxidation of SIRT2 during increased oxidative stress of hyper-inflammation decreases its enzymatic activity and NFĸB p65-deacetylation function [105]. The effect of oxidation of SIRT2 on deacetylation of PKM2 to modulate its tetramerization during hyper- and hypo-inflammation needs further elucidation. Lastly, under nutritional deprivation conditions, the liver uses the glycogenolysis and gluconeogenesis pathways. Phosphoenolpyruvate carboxykinase 1 (PEPCK1) is the rate-limiting enzyme in this process. SIRT2 deacetylates and stabilizes PEPCK1 to increase its activity [106].

SIRT2 represses adipocyte differentiation by deacetylating FOXO1, which then translocates to the nucleus to repress adipogenesis by repressing PPARγ. Conversely, SIRT2 deficiency enhances FOXO1 acetylation, allowing it to retain it in the cytosol to be phosphorylated and thus ultimately unable to enter the nucleus to repress PPARγ [92]. Lipogenesis, described as a process of de novo fatty acid synthesis from excess carbohydrate consumption, also occurs during the acute hyper-inflammatory phase [107]. This anabolic pathway includes activating cytosolic ATP-citrate lyase (ACLY), an enzyme that catalyzes the conversion of citrate to acetyl-CoA, which diverts fatty acids from entering the mitochondria and activates fatty acid synthase as a rate-limiting building block in lipogenesis [108]. SIRT2 modulates ACLY activity by deacetylation and ubiquitination followed by degradation. Thus, SIRT2 regulates lipogenesis during over nutrition [109]. Krishnan et al. demonstrated the expression and activity of hypoxia inducing factor (HIF)-1α in the adipose tissue. HIF-1α negatively regulates the SIRT2-PGC-1α regulatory axis, which in turn inhibits fatty acid β oxidation, leading to increased adiposity [93].

SIRT2 activation with increased NAD+ availability supports the hypo-inflammatory phase and induces endotoxin tolerance in monocytes and in septic mice. To do this, SIRT2 translocates to the nucleus under cellular stress and like SIRT1 deacetylates NFĸB p65 [80,110,111]. Others and we have shown that SIRT2 deacetylates and deactivates NFĸB p65 during acute inflammation [13,110].

We showed that while SIRT1 is important during hypo-inflammation in lean mice (described above), it is not expressed during hypo-inflammation of obese mice with sepsis. In obese mice with sepsis, increased SIRT2 protein levels prolong hypo-inflammation instead; SIRT2 is the most abundant of sirtuins in the adipose tissue [13]. Specifically, we showed that SIRT2 expression decreases during hyper-inflammation and increases during the hypo-inflammatory phase in obesity with sepsis via direct deacetylation of NFĸB p65. We also discovered that SIRT2 inhibition using SIRT2 inhibitor AK-7 during the hypo-inflammatory phase reversed hypo-inflammation via endothelial and circulating cell activation and increased survival in these mice [13]. The striking dichotomy between SIRT1 and SIRT2 in nuclear functions and physiologic effects in lean vs. hyper nutrition with obesity is not fully understood. This critical gap in understanding sepsis inflammation is important to fill.

We have also shown that SIRT2 knock out mice exhibit increased microvascular inflammation, while SIRT2 overexpression is associated with decreased microvascular inflammation hyper-inflammatory phase in sepsis mice [112]. Evidence suggests that SIRT2 knock out mice show increased bacterial clearance in sepsis [113].

What causes the SIRT2 expression to decrease during hyper-inflammation? We have recently shown that the relationship between obesity with sepsis and SIRT2 is a two-way street. While SIRT2 modulates sepsis inflammation, sepsis inflammation and oxidative stress also regulate SIRT2. Four cysteines flank the zinc tetrathiolate motif of all sirtuins; this is important for the structural and functional stability of the sirtuin molecule. We showed that direct oxidation of these cysteines during oxidative stress of hyper-inflammation modulates the enzymatic and deacetylation activity of SIRT2 [105].

### 4.3. SIRT3

SIRT3 is one of the three mitochondrial deacetylases, namely SIRT 3, 4 and 5 [114]. While it is known to localize in mitochondria, a great deal of controversy exists regarding a possible location in the nucleus [115,116,117]; its best-known biochemical and physiologic functions are mitochondrial. SIRT3 is linked to longevity in humans; specific mutations in the enhancer region of the *SIRT3* gene were associated with increased life span [118]. Other physiologic functions of SIRT3 in inflammation include myocardial dysfunction in diabetes, insulin resistance, lung fibrosis and kidney injury responses [119,120,121,122,123].

SIRT3 deacetylase activity alters fatty acid metabolism and mitochondrial biogenesis [118]. SIRT3 expression selectively increases in brown adipose over white adipose tissue during cold exposure and caloric restriction. Thus, SIRT3 supports catabolic thermogenesis by fatty acid oxidation via PGC-1α-induced support of lipolysis; UCP1 supports increased heat rather than ATP formation in brown adipocytes [124]. SIRT3 also modulates substrate selection under stressful conditions. During caloric restriction and unlike SIRT1, decreased SIRT3 expression in skeletal muscle disrupts glucose oxidation by directly inhibiting pyruvate dehydrogenase (PDH) activity. Moreover, this SIRT3 deficiency is associated with substrate switch from glucose oxidation to fatty acid utilization via PDH activity in skeletal muscle [125]. Thus, SIRT3 deficiency seems to influence the metabolic flexibility of skeletal muscle under stressful conditions, which likely impacts inflammation.

Decreased SIRT1/SIRT3 protein levels correlate with increased glycolysis and its adverse effect on cardiac dysfunction during early sepsis. Inhibiting glycolysis by 2-deoxyglucose, a hexokinase-2 inhibitor, prevents cardiac dysfunction and glycolysis along with increased SIRT1/SIRT3 levels during early sepsis [126]. This suggests that SIRT3 support of fatty acid oxidation during sepsis tolerance is cardio protective. Moreover, SIRT3 knock out mice show increased acute kidney injury during sepsis [115]. Additionally, SIRT3 modulates pericyte loss and vascular dysfunction during early sepsis [127].

We studied the role of SIRT3 during the late/hypo-inflammatory phase of sepsis. We demonstrated sustained increase in SIRT1 and SIRT3 protein levels during the hypo-inflammatory phase of sepsis in which fatty acid oxidation replaces glycolysis as the predominant fuel source. Mechanistically, SIRT1 and SIRT3 repress mitochondrial OXPHOS and reduce mitochondrial biogenesis during hypo-inflammation and endotoxin tolerance. Under the control of SIRT1 and SIRT3, these immune cells become dependent on fatty acid oxidation [9]. Moreover, SIRT1 inhibition during hypo-inflammation reversed these changes, supporting its proximal effects on the switch in lean mice [9].

Taken together, SIRT3 joins SIRT1 and SIRT6 (discussed below) in protecting homeostasis, but prolonging the potentially lethal tolerance and immunometabolic paralysis of human and mouse sepsis [20]. What then might physiologically counter the tolerance prolongation?

### 4.4. SIRT4

SIRT4, another mitochondrial sirtuin, is expressed in kidney, heart, brain, liver and monocytes [128]. SIRT4 was first emphasized for its ADP-ribosylation function, used in ADP-ribosylation of glutamate dehydrogenase (GDH), which converts glutamate to α-ketoglutarate in mitochondria. SIRT4 represses the enzymatic activity of GDH, thereby limiting glutamate/glutamine metabolism to generate ATP [128]. It received special attention because some cancer cells, in addition to aerobic glycolysis, use glutaminolysis to sustain cell proliferation [129]. SIRT4 was thus established as being critically important for a cross-talk between glycolysis and glutaminolysis during sustained proliferation of cancer cells [129]. More recently, other enzymatic activities such as lipoyl- and biotinyl-lysine modifications in regulating the pyruvate dehydrogenase complex were identified; the importance of these activities relative to each other remains unknown [130].

The role of SIRT4 in inflammation and sepsis is largely unexplored. Evidence suggests that expressed very late during hypo-inflammation, SIRT4 is a physiological mechanism that counters hypo-inflammation and is involved in sepsis resolution [81]. SIRT4 expression decreases during early inflammation in endothelial cells. Similarly, SIRT4 knock down is associated with increased inflammatory response, while SIRT4 overexpression decreases inflammatory response to LPS [82]. Mechanistically, SIRT4 increases glycolysis and glucose oxidation by diminishing the activity of PDH [130]. It also reverses fatty acid oxidation in endotoxin-tolerant THP-1 monocytes to glucose oxidation and increases expression of fatty acid synthase. SIRT4 expression increases in monocytes in vitro and in human sepsis blood monocytes that have been reprogrammed from hyper-inflammation to hypo-inflammation and endotoxin tolerance [82]. Thus, SIRT4 deacetylation [131] and ADP ribosylation of proteins’ [128] transferase properties counter the effects of mitochondrial SIRT3. Thus, SIRT4 seems to be a candidate for physiologically breaking in vitro endotoxin tolerance seen during sepsis; the in vivo role in sepsis needs further elucidation.

### 4.5. SIRT6

Nuclear SIRT6 is a master glucose homeostat that controls the glucose component of the endotoxin tolerance axis, in which SIRT1 predominantly controls the lipid fatty acid oxidation (FAO) axis. HIF1-α and TORC are the “yin” and SIRT 6 and 1 the “yang” of the “yin/yang” [132]. In the nucleolus, SIRT6 associates with telomeres; SIRT6-deficient cells show abnormal end-to-end chromosomal fusion and premature senescence similar to that seen in Werner syndrome with premature ageing [133,134].

SIRT6 deficiency is associated with profound metabolic dysregulation. SIRT6-deficient mice, while born normally, develop acute degenerative processes including severe loss of subcutaneous fat, lymphopenia and osteopenia and die within the first month of life of acute onset hypoglycemia [135]. Glycolytic enzymes are modulated by acetylation/deacetylation in the macrophages [136]. SIRT6 deficiency increases GLUT1 expression on the cell surface (increasing glucose uptake,) as well as upregulates the expression of several of the key glycolytic enzymes causing hypoglycemia in mice. This indicates that SIRT6 transcriptionally represses these key glycolytic enzymes. Moreover, SIRT6 acts as a corepressor of transcription factor HIF1-α to regulate nutrient stress response [135].

SIRT6 exerts anti-inflammatory actions, like SIRT1 and SIRT2, by deacetylating and deactivating NFĸB p65 to promote hypo-inflammation and endotoxin tolerance in immune cells [137]. SIRT6-deficient cells show hyperacetylation of H3K9 at target promotors associated with increased NFĸB p65 promotor occupancy to enhance NFĸB-dependent gene expression and pro-inflammatory phenotype [7,137]. Conversely, SIRT6 overexpression suppresses NFĸB-dependent gene expression in response to TNF-α stimulation and decreased local and systemic inflammation [138]. SIRT6 supports the switch from hyper- to hypo-inflammatory response in sepsis inflammation by countering NFĸB p65 and HIF1-α-dependent glycolysis during hyper-inflammation, while SIRT1 couples to PGC-1 to increase fatty acid oxidation as hypo-inflammation and endotoxin tolerance develop; specifically, SIRT1 and SIRT6 coordinate the immunometabolic switch during sepsis [7]. What informs this coupling? Others and we found that direct oxidation of SIRT1 [139] and SIRT6 cysteine thiol oxidation reversibly inactivate the zinc tetra thiolate motif. We find that these cysteines on SIRT6, similar to that seen in SIRT2 (discussed above), are inactivated during hyper-inflammation and reactivated during the hypo-inflammatory phase with antioxidant pathways. This reciprocal nature of oxidation and anti-oxidation modulates glucose and fatty acid homeostat during immuno-metabolic programming [140]. Whether SIRT 3 and 4, as well as the other SIRT family members are directly redoxed is an important area for investigation, since all SIRTs share the zinc-linked cysteine tetrad.

### 4.6. SIRT5 and SIRT7

Sirtuin 5 is one of the least understood sirtuins. Although described initially as a deacetylase, SIRT5 was subsequently shown to be an efficient protein lysine desuccinylase and demalonylase [141,142,143,144]. Evidence suggests that primarily located in mitochondria, SIRT5 also exerts its activity in cytosol [145]. While expressed in all tissues, SIRT5 appears enriched in brain and heart tissue. The role for SIRT5 as a metabolic and inflammatory regulator is still emerging. The literature indicates that SIRT5 regulates ischemia reperfusion injury in heart and brain and has a role in cancer [141,146,147].

Various mechanisms are implicated in SIRT5-induced post-translational modifications in cardiac ischemia/reperfusion (I/R) injury. SIRT5 deacetylates STAT 3 to modulate its function and thus affecting mitochondrial pyruvate metabolism [146,148]. SIRT5 also regulates lysine demalonylation to increase malonyl-CoA, a TCA cycle intermediate that inhibits CPT1 and mitochondrial fatty acid. This may contribute to efficient energy utilization and cardiac I/R injury modulation, as well [143,146]. In brain I/R injury, evidence suggests that SIRT5 regulates blood brain barrier function via degradation of occludin. In this model, SIRT5 deletion is associated with decreased infarct size [147].

What regulates SIRT5 expression? Evidence suggests that SIRT5 is under transcriptional control of PGC-1α; PGC-1α knock down attenuates, while overexpression increases SIRT5 mRNA and protein levels. However, under fasting conditions, while induction of PGC-1α is detected, a corresponding increase in SIRT5 does not occur. It also appears that under these conditions, AMPK downregulates SIRT5 expression. Therefore, under fasting conditions, in a complex relationship, while increased PGC-1α is poised to increase, AMPK induction downregulates SIRT5 expression [149].

Emerging evidence suggest a role for SIRT5 during the hypo-inflammatory phase of sepsis. In contrast with SIRT1 and SIRT2, SIRT5 expression is decreased during the hypo-inflammatory phase of sepsis. Interestingly, SIRT5 localizes itself in the cytoplasm and interacts with SIRT2 to antagonize it. As a result, SIRT5, in effect, decreases interaction between SIRT2 and NFĸB p65 to effectively increase its acetylation; in turn, enhancing the pro-inflammatory response [145]. Although the exact mechanism of this interaction remains unknown, a picture of a rather complex relationship between different members of the sirtuin family starts to emerge.

With its nucleolar localization, SIRT7 is increasingly known for its role in genomic stability [150]. SIRT7 is recruited to DNA double-stranded breaks (DSBs) to catalyze desuccinylation of lysine 122 on H3 to promote chromatin condensation and repair of DSBs [150,151]. SIRT7 deletion shows impairment of DNA damage repair via modulation of lysine 18 acetylation and reduces lifespan with progeroid-like features in mice [151,152,153].

Similar to other members of the family, SIRT7 participates in metabolic regulation of cells under stressful conditions. Evidence suggests that SIRT7 suppresses endoplasmic reticulum stress (ER stress) via acting as a cofactor of Myc for transcription repression to modulate ER stress; SIRT7 knock out mice show chronic hepatosteatosis resembling fatty liver disease in humans. Reintroduction of SIRT7 represses ER stress and reverts fatty liver disease [151,154].

The role of SIRT7 in inflammatory conditions and particularly that in sepsis and septic shock is not very well defined yet. However, there is a growing body of evidence regarding numerous targets of SIRT7 suggesting a crucial role for SIRT7 in genomic stability and metabolic response of cells under stressful conditions. With that, SIRT7 will emerge as an important player in inflammatory conditions, as well [155].

## 5. Sirtuin Modulation as Potential Therapeutic Targets?

Sirtuins guard immuno-metabolic response during acute inflammation. Departure from homeostasis, seen in sepsis and septic shock, occurs via dysregulation of sirtuin expression; sirtuin deficiency during hyper-inflammation and sustained sirtuin expression during hypo-inflammation outlined in the sections so far pose unique opportunities for phase-specific therapies.

### 5.1. Sirtuin Modulation during Hyper-Inflammation

We have shown that sirtuin overexpression is associated with attenuation of the hyper-inflammatory phase in rodent sepsis with increased survival [112]. Evidence suggests resveratrol pre-treatment attenuates sepsis-related inflammatory response in mice [71,79,156]. We have shown that pre-treatment with resveratrol in obese mice attenuates the hyper-inflammation and improves survival. Mechanistically, resveratrol attenuated microvascular inflammation and adhesion molecule expression via direct deacetylation of NFκB p65 in endothelial cells [79].

### 5.2. Sirtuin Modulation during Hypo-Inflammation

Over 60% of sepsis-related mortality occurs during late sepsis. While it would be ideal to prevent sepsis, it is not always feasible; patients may not present to the healthcare facilities early in the course of their disease. Late sepsis/hypo-inflammation is characterized by sustained increase in sirtuin expression. Sirtuin inhibition during this phase seems to be a therapeutic option [19]. We have shown that sirtuin inhibition during the hypo-inflammatory phase of sepsis improves survival. Specifically, SIRT1 inhibition using EX-527 during the hypo-inflammatory phase of lean mice with sepsis improved bacterial clearance, shortened hypo-inflammation and improved survival in these mice. Interestingly, we observed that while the hypo-inflammatory phase was prolonged in obese mice compared to lean, SIRT1 did not play a crucial role. We observed that, instead, SIRT2 expression increased in obese mice with sepsis hypo-inflammation. SIRT2 inhibition using AK-7 compound during the hypo-inflammatory phase of obese mice with sepsis reversed hypo-inflammation via activation of immune cells and endothelial cells, as well as improved survival [13].

However, SIRT1 inhibition during early sepsis decreased survival in lean mice [19]. Thus, knowing the exact phase of sepsis is of critical importance before using small molecular inhibitors of sirtuins. Similarly, we observed decreased survival in obese mice with SIRT1 inhibition during hypo-inflammation of sepsis in obese mice [13]. Thus, different members of the sirtuin family of proteins, while similar in many of their immuno-metabolic properties, seem to have distinct biological and context-dependent properties that warrant further elucidation. In addition, the interactions between different members of sirtuin family members, like those seen between SIRT5 and SIRT2, need further elucidation. While still somewhat premature, sirtuin modulation in a phase-specific manner seems to have promise in sepsis therapy.

## 6. Conclusions

Sepsis transitions from glycolysis-dependent hyper-inflammation to a fatty acid-dependent hypo-inflammatory phenotype. Sirtuins are integral to this immuno-metabolic transition between the two phases. Phase-specific modulations of sirtuins are attractive potential therapeutic targets in sepsis.

## Figures and Tables

**Table 1:** The role of different sirtuins in various immune cells during sepsis.

Sirtuin	Immune Cell Type	Mechanism of Action
**SIRT1**	Monocytes	Direct NFκB p65 deacetylation and the HIF-1α and PGC-1α pathway [14]
	Macrophages	Phenotypic shift from activator to suppressor cells [19]
	Lymphocytes	Suppression of pro-inflammatory cytokine expression [71]
	Endothelial cells	Direct NFκB p65 deacetylation: attenuation of pro-inflammatory adhesion molecule expression [79]
**SIRT2**	Macrophages	Direct NFκB p65 deacetylation and polarization to suppressor phenotype: STAT6/GATA3 signaling cascade [80]. Direct NFκB p65 deacetylation: attenuation of pro-inflammatory cytokine expression [13]
**SIRT3**	Monocytes	Mitochondrial biogenesis and increased oxidative phosphorylation to sustain hypo-inflammation [9]
**SIRT4**	Monocytes	Resolution of hypo-inflammatory phase; restoration of glucose oxidation via PDC activity and SIRT1 repression [81]
	Endothelial cells	Attenuation of pro-inflammatory cytokine and adhesion molecule expression via blocking of nuclear translocation of NFκB p65 [82]
**SIRT6**	Monocytes	Decreases glucose oxidation and glycolysis during the hypo-inflammatory phase via epigenetic repression of HIF-1α [7]

## Abbreviations

Sir2	Silent mating-type information regulator 2
SIRT	Sirtuin
ADP	Adenosine diphosphate
ATP	Adenosine triphosphate
NAD	Nicotinamide adenine dinucleotide
NAMPT	Nicotinamide phosphoribosyltransferase
GLUT	Glucose transporter
PDC	Pyruvate dehydrogenase complex
Acetyl-CoA	Acetyl coenzyme A
NADPH	Nicotinamide adenine dinucleotide phosphate
ROS	Reactive oxygen species
TCA	Tricarboxylic acid
CPT1	Carnitine palmitoyltransferase 1
STAT6	Signal transducer and activator of transcription 6
PPAR	Peroxisome proliferator-activated receptor
PGC	Peroxisome proliferator-activated receptor gamma coactivator
FOXO1	Forkhead box protein O1
HIF	Hypoxia inducible factor
LD	linear dichroism
UCP1	Uncoupling protein 1
PDH	Pyruvate dehydrogenase
ACLY	ATP-citrate lyase
PEPCK1	Phosphoenolpyruvate carboxykinase 1
OXPHOS	Oxidative phosphorylation
NFĸB	Nuclear factor kappa B
TNF	Tumor necrosis factor
H3K9	Histone 3; lysine 9
CD36	Cluster of differentiation 36
AMPK	AMP-activated protein kinase
